# Tropospheric Photochemistry of 2-Butenedial: Role of the Triplet States, CO and Acrolein Formation, and the Experimentally Unidentified Carbonyl Compound—Theoretical Study

**DOI:** 10.3390/molecules29030575

**Published:** 2024-01-24

**Authors:** Andrea Maranzana, Glauco Tonachini

**Affiliations:** Dipartimento di Chimica, Università di Torino, Corso Massimo D’Azeglio, 48, I-10125 Torino, Italy

**Keywords:** 2-butenedial, triplet states, ISC, CO loss, DFT, reaction mechanism

## Abstract

Solar irradiation of 2-butenedial in the lower troposphere mainly produces isomeric ketene-enol (a key intermediate product), furanones, and maleic anhydride, the formation pathways of which were investigated in a previous study. The other main products were carbon monoxide and an experimentally unidentified carbonyl compound. This was the subject of the present study. The oxidative reaction mechanisms were studied using DFT calculations. Water intervention is found essential. Its addition and subsequent water-assisted isomerizations (an ene-*gem*-diol/enol and a carboxylic acid/enol form), followed by cyclization, lead to an interesting cyclic carbonyl compound, but this pathway appears to be rather energy demanding. An alternative implies water cooperation in a ketene-enol + carboxylic acid/enol addition that gives the relevant anhydride. The anhydride is proposed as a candidate for the experimentally unidentified carbonyl product. Regarding CO and acrolein formation, the role of the triplet states, as defined by the probability of intersystem crossing from the excited singlet state S_1_ to T_2_ and T_1_, is discussed. The T_1_ photolysis pathway connecting butenedial to propenal + CO was then defined.

## 1. Introduction

Unsaturated 1,4-dicarbonyls are generated through chemical reactions involving aromatic hydrocarbons [1,2], and OH groups, as well as biomass combustion [3]. The oxidation processes that lead to the formation of these compounds have been studied theoretically, and they have been demonstrated to result in the formation of carbonyl compounds (as referenced in [4]). Specifically, dicarbonyl compounds are prone to rapid photochemical degradation when exposed to atmospheric conditions [4].

The study of the photolysis and OH reaction of *Z*- and *E*,*E*-2,4-hexadienedial was conducted by Barnes et al. [5]. The authors found that the reaction with OH radicals was typically a secondary channel, and only during the summer could the OH radical concentrations compete with photolysis as a reaction pathway. The identified photolysis products were 2-formyl-2*H*-pyran, 3,4-diformyl-cyclobutene, and 2-butenal-4-yl-ketene.

Reaction of *E*,*E*-2,4-hexadienal with hydroxyl radical was also theoretically studied by Sun et al. [6]. The authors concluded that E-butenedial was the dominant product of hexadienal photooxidation.

The rate constant for the reaction *E*-butenedial + OH was assessed by Martín et al. as 3.45 ± 0.34 × 10^−11^ molec cm^3^ s^−1^, mainly due to the H abstraction process, whereas the photolytic rate coefficient was 3.6 ± 0.03 × 10^−4^ s^−1^. Thus, under typical atmospheric conditions, the photolysis reaction is considered the major atmospheric sink for *E*-butenedial [7].

In a recent study by Newland et colleagues [8], the photochemistry of 2-butenedial and 4-oxo-2-pentenal was investigated in an outdoor photoreactor (Euphore) in the presence and absence of OH radicals. FTIR spectroscopy was used to detect the major products of 2-butenedial degradation in situ. The results revealed that 3*H*-furan-2-one, maleic anhydride, CO, and an unidentified carbonyl compound were the primary byproducts of this reaction. Additionally, minor amounts of maleic anhydride, formaldehyde, glyoxal, 5*H*-furan-2-one, and acrolein were also detected. Previous studies by Marshall et al. [9] and Back and Parsons [10] also reported the formation of CO, CO_2_, and C_2_H_2_ products upon photolysis of butenedial.

In a study conducted by Tang and Zhu [11], they used photolysis to investigate the decomposition of butenedial at various wavelengths, including 193, 248, 280, 308, 351, 400, and 450 nm. Acrolein and 3*H*-furan-2-one were detected as products of the reaction, but no HCO radical was detected in the region of 280–450 nm. In a previous study by Bierbach et al. [12], the FTIR spectra detected 3*H*-furan-2-one and maleic anhydride after irradiation at 320 nm < λ < 480 nm. They also proposed a reaction mechanism for the formation of 3*H*-furan-2-one via a diradical or zwitterionic intermediate. The production of the ketene-enol intermediate was hypothesized to occur via a Norrish Type II process [13,14] and intramolecular H-atom transfer [15]. Interestingly, Newland et al. [8] observed that ketene-enol was formed when 2-butenedial was irradiated by solar light and rapidly disappeared in the dark, which was related to the formation of furanones and maleic anhydride. The authors suggested that 3*H*-furan-2-one was formed by the cyclization of ketene-enol.

Other researchers have also observed a similar reaction in studies of the aromatic compound phthalaldehyde [16,17,18,19]. When o-phthalaldehyde was exposed to UV light, phthalide and a dimeric product were produced. Scaiano et al. concluded that the reaction did not involve triplet states because they had a longer lifetime, and instead involved a biradical with a short lifetime (1.6 μs) that was produced by intramolecular hydrogen transfer. They suggested that ketene-enol and a cyclic enol were intermediate products in the reaction [16].

Blancafort et al. [17] used multireference calculations to study the phototautomerization of o-phthalaldehyde, and they found that ketene-enol was formed by H transfer in the S_1_ state, followed by a conical intersection S_1_/S_0_ that led to the intermediate in the ground state. They did not consider the role of triplet states.

Fröbel et al. [18] used femtosecond-stimulated Raman spectroscopy and quantum calculations to study the formation of phthalide from o-phthalaldehyde. They also identified ketene-enol as an intermediate in the reaction, formed by relaxation from the lowest excited singlet state to the ground state. However, they found that ISC (S_1_/T_1_) was very efficient (≈5 ps), suggesting that the triplet path may also be relevant.

He et al. [20] utilized a complete-active-space self-consistent field (CASSCF) and density functional theory to investigate the photochemistry of butyrophenone. They identified all the minima, transition states (TS), and minimum energy crossing points among the S_1_, T_1_, and T_2_ states. The study revealed that the S_1_/T_1_ intersystem crossing (ISC) occurred at a low rate, while the S_1_/T_2_ ISC was a rapid process. Moreover, the T_2_/T_1_ internal conversion was expected to be extremely fast. Overall, the S_1_/T_1_ ISC was found to be fast (approximately 10^11^ s^−1^), and the 1,5-H shift (Norrish type II process) occurred in the T_1_ state, leading to the formation of a triplet 1,4-biradical intermediate.

Rowell et al. [21,22] investigated 20 carbonyl compounds, excluding 2-butenedial. The photochemical process can occur in either T_1_ or S_1_ states, depending on the reactants and the S_1_ energy threshold, which is typically the Norrish type II reaction’s threshold. When the S_1_ threshold is high, the T_1_ state becomes dominant. For some α,β-unsaturated carbonyls, photoisomerization occurs in the S_1_ state and then crosses to S_0_ via the S_1_/S_0_ conical intersection. When the S_1_ energy barrier is high, an ISC to T_1_ transition takes place. With saturated carbonyls, photolysis in the T_1_ state is competitive or dominant. The α-bond cleavage photolysis of 20 atmospherically relevant carbonyls is possible in the T_1_ state or on an internally hot S_0_ state [23].

In the present study, we consider the photo-oxidation of 2-butenedial, in particular the possible role of triplet states, and the formation of carbon monoxide. We will also try to clarify the nature and formation mechanism of the experimentally unidentified carbonyl compound that flanks the formation of the other main oxidation products, already considered in our previous study.

## 2. Results

### 2.1. Assessment of a Suitable Computational Level to Identify the Unknown Carbonyl Product

Newland et al. reported that “a carbonyl product with a C=O stretching absorption band around 1819 cm^−1^ was also observed as a major product but could not be identified” [8]. Prompted by their observation, we intended to carry out a vibrational analysis for some molecules that might be deemed reasonable candidates for being the unknown carbonyl compound. Two requirements obviously need to be satisfied. In addition to their C=O stretching frequency νC=O falling close to the above value, the reaction pathway through which these products are obtained should present free energy barriers that are not too high. To this end, we have first tested some DFT computational levels suitable to confidently assess the value of νC=O, and we will discuss later (Section 2.2) the possible nature of the candidate molecules and the relevant reaction pathways leading to them.

**Figure 1:**
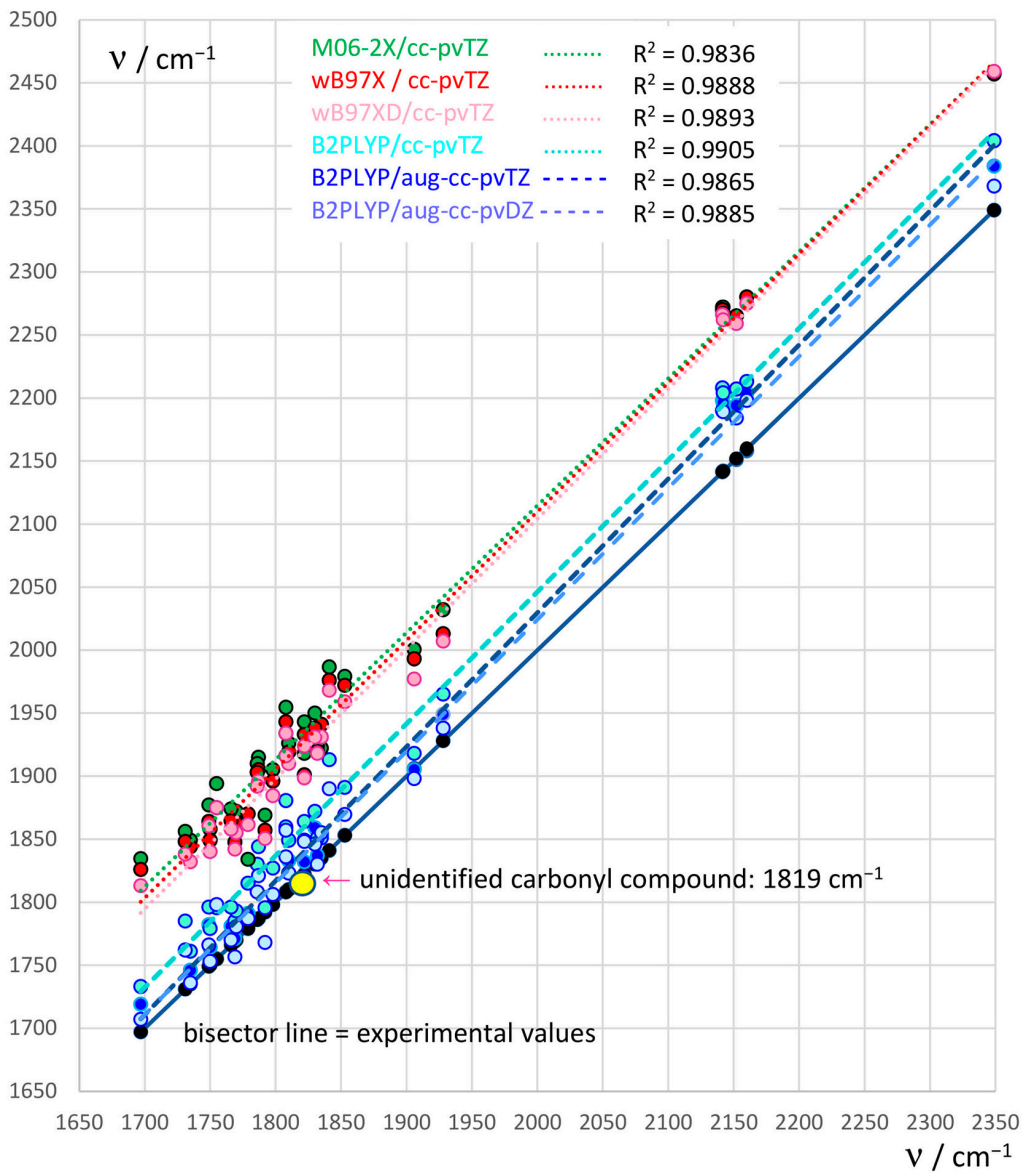
C=O stretching frequencies νC=O /cm^−1^. The black bisector line collects the experimental data (black dots; yellow dot: the unidentified carbonyl compound). Other colored dots correspond to νC=O frequencies calculated by different DFT methods and basis sets. B2PLYP/aug-cc-pVTZ, blue dashed line; B2PLYP/cc-pVTZ, turquois dashed line; B2PLYP/aug-cc-pVDZ light blue dashed line. Comparison with the dotted lines: M06-2X/cc-pVTZ, green; ωB97XD/cc-pVTZ, pink; ωB97X/cc-pVTZ, red.

Four DFT functionals, M06-2X [24], ωB97X [25], ωB97XD [26] and B2PLYP [27], were tested for a pool of 31 (25, or 18, depending on the basis set, in the gas phase) vibrational frequencies of carbonyl compounds for which the experimental datum is available. Their performance is shown in Figure 1, where the black bisector line corresponds to the experimental νC=O values. Almost all of them overestimate the νC=O frequency, however by different amounts. The best performance is exhibited by B2PLYP, in conjunction with the Dunning’s basis set aug-cc-pVTZ (blue dashed line, regression on the data for 19 molecules). It proves to be the most accurate, with a mean error of 23 cm^−1^. The basis set was however tested also without diffuse functions (aug) because for the largest of our candidate molecules, the calculation with aug-cc-pVTZ proved unfeasible. The performance of the B2PLYP/cc-pVTZ (on 31 molecules) is shown by the turquois dashed line, and the mean error is now 40 cm^−1^. As an alternative (suggested by one referee), the computational level B2PLYP/aug-cc-pVDZ was also explored (light blue dashed line). Unexpectedly, when compared to the more flexible aug-cc-pVTZ, using its same set of 19 data, this smaller basis set gives a better performance (mean error: 14 cm^−1^). A few estimates of νC=O resulted with a negative error: this fact contributes in part to its apparently better performance, since it shifts the regression line downwards. We consider this result to some extent suspicious, not to be taken too literally; however, this computational level could be used for treating (with due caution) our largest candidate molecule. The performance of the other three functionals (with the cc-pVTZ basis set) is not as good compared to B2PLYP, as can be seen in Figure 1. Here, the regression lines are grouped in two sets: the lowest set, more accurate, pertaining to B2PLYP results with the said three different basis sets, and the top set, less accurate, to the other three functionals with cc-pVTZ. The closeness of the lines related to ωB97X (red data) and ωB97XD (pink data) indicates that dispersion forces play here a limited role. It is noteworthy that the obtained regression lines present R^2^ values close to 0.99 and are almost parallel to the bisector line. We feel that the procedure of applying mean errors derived from this analysis to correct the computational values coming from the vibrational analysis on a test molecule will be acceptably safe, though not exempt from limitations regarding the computational level or the number and quality of IR data taken into account.

### 2.2. Gas-Phase Reaction Pathways Leading to Candidate Products

In our previous paper on butenedial photo-oxidation [24], the role of water molecules was found to be crucial in allowing us to define viable pathways to main products experimentally detected, namely 3*H*-furan-2-one, 5*H*-furan-2-one, and maleic anhydride. In fact, ketene is known to react with H_2_O. Nguyen et al. [28] carried out in 2013 theoretical calculations on the ketene/water system. On the basis of that study, the authors concluded that the formation of acetic acid quite likely occurs in the high-temperature combustion of biomass, but that the rate of formation should be negligible under ambient atmospheric conditions. In a paper published in the same year, Kahan et al. [29] observed that the gas-phase products are acetic acid, acetic acid dimer, and acetic anhydride. They report that the time-dependence of product formation supports a reaction mechanism in which ketene hydrates to form acetic acid, which then can combine with ketene to form acetic anhydride. These authors concluded, in contrast with the results of Nguyen et al., that ketene can undergo hydration under atmospherically relevant temperatures and relative humidities. It has also been reported that a water molecule easily adds to ketene to give 1,1-dihydroxyethene, while acetic anhydride is produced by the reaction of acetic acid with ketene.

We have pursued two lines of search (Section 2.2.1 and Section 2.2.2) to ascertain if some re-action steps can produce a carbonyl compound having C=O stretching frequencies close to that observed by Newland et al. [8].

#### 2.2.1. Reaction Pathways to One Cyclic Candidate Product

**Scheme 1:**
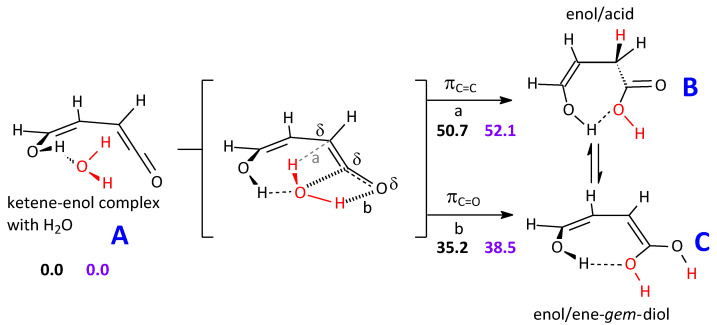
Possible steps in the formation of ketene-H_2_O adducts in the gas phase. The addition to the carbonyl group is favored. Only one explicit water molecule is shown.

Addition of one water molecule to ketene-enol can form, in principle and starting from the complex **A**, two different products: B and C. An enol/carboxylic acid **B** intermediate would result from an addition of water to the C=C bond. Otherwise, an enol/ene-*gem*-diol **C** intermediate would be the product of an addition to the C=O bond. The latter acetal is believed to be the product of the preferred water addition mode to ketene [28,30,31]. In Scheme 1, we report the energy and free energy barriers pertinent to the process of water addition, as sketched by involving just one water molecule, which becomes embedded in the intermediate products **B** and **C**. Though the order of the barrier heights corresponds to the preference indicated in the references just reported, it is obvious that the barriers are prohibitively high.

**Scheme 2:**
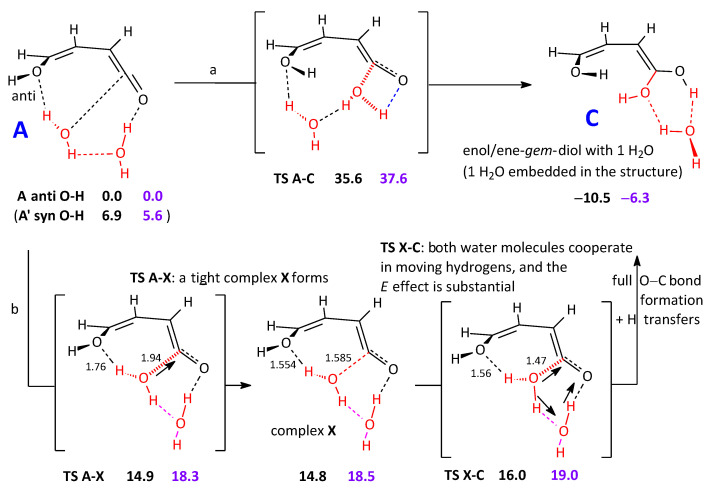
Addition of one water molecule (red) to the carbonyl group of the ketene-enol intermediate to give an enol/ene-*gem*-diol intermediate. Role of a second water molecule (red) in lowering the free energy barrier. Complex **X** does not appear to have a role in terms of *G*. Energy differences: black figures. Free energy differences: purple figures.

Therefore, both these reaction steps could not take place without the intervention of other factors. One could be the involvement of other water molecules in a cooperative effect. Given the estimated ratio 100:1 for water and the reacting system [32], it seems reasonable to surmise that one or two water molecules can interact at any moment with the intermediate at hand. This point is important, because a supporting assistance can make feasible some gas-phase reaction steps that would otherwise present high barriers. The initial water addition step to the ketene-enol in **A**–**C** illustrates this point well (Scheme 2). Actually, sheer assistance by a second interacting water molecule has a negligible effect (top line), and the free energy barrier remains substantial (37.6 kcal mol^−1^). The effect is limited because this second water interacts but is not directly involved in the hydrogen shifts. If, on the other hand, the second H_2_O is openly part of the bond breaking/ bond formation step, a truly cooperative effect is apparent, and the free energy barrier drops to 19.0 kcal mol^−1^ (bottom line). It can be noticed that the role of the intermediate tight complex, labeled **X**, vanishes in terms of *G*, and only one step apparently connects **A** to **C**. It can also be observed that the O–H hydrogen, set in anti-position with respect to the double bond in **A**, spontaneously turns to *syn* at some point in the TS search. We can comment in passing that the attack of water to the C=C requires (at the same computational level) overcoming an *E* barrier of 30.1 kcal mol^−1^, 32.0 for *G*, a second water molecule cooperating.

**Scheme 3:**
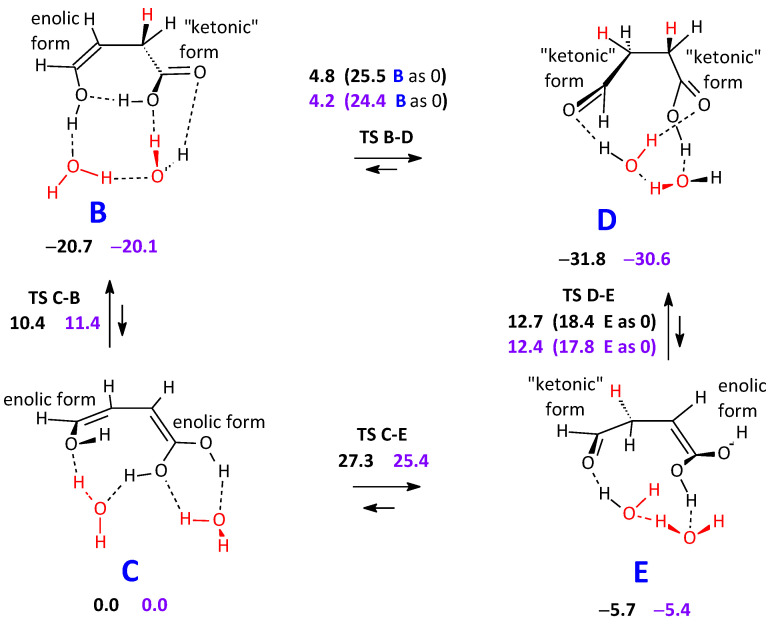
Keto-enol tautomeric equilibria connecting the initial adduct **C** to its isomers **B**, **D**, and **E**. Two cooperating water molecules assist (red). The **C**–**B**–**D** transformation appears to be to some extent more likely. Energy differences: black figures. Free energy differences: purple figures.

Once **C** is formed, with the likely assistance of two cooperating water molecules, three other intermediates are related to it by keto-enol tautomerism (Scheme 3). **C** will be related to **B** and, through it, to an aldehyde/acid intermediate **D**; then, both **C** and **D** will be related to an aldehyde/ene-gem-diol intermediate **E**. The **C**–**B**–**D** transformation appears to be somewhat more likely, though rather energy demanding. In the initial step leading from **A** to **C**, one water molecule had become embedded in the structure. Now, two extra water molecules can possibly make these keto-enol transformations easier.

The effect of introducing a third water molecule has also been explored. On the one hand, it should be fully cooperated into the hydrogen shifts, as the other two are. On the other hand, the geometry of the process requires it to accommodate the three water molecules in a limited space. The outcome, while fulfilling these requirements, puts the *G* barrier for the **B**–**D** step at 23.8–23.9 (two different transition structures found) kcal mol^−1^. The effect of the third interacting water is therefore not too supportive in making the **C**–**B**–**D** transformation easier. The **C**–**E** free energy barrier results in even 31.0 kcal mol^−1^ high.

**Scheme 4:**
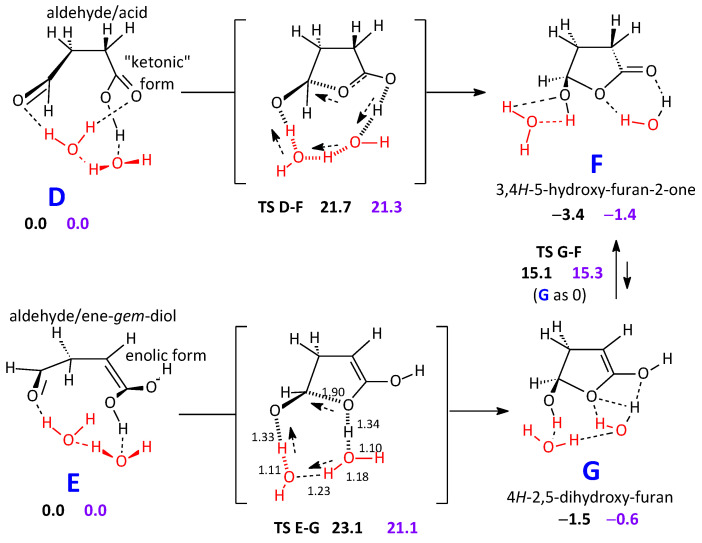
Ring closure step from **D** and **E**. Keto-enol tautomeric equilibrium between the cyclic products **G** and **F**. All steps can converge on the lactone **F**, possible candidate to be the “unidentified carbonyl compound”. Energy differences: black figures. Free energy differences: purple figures.

From the intermediates **B** and **C** involved in the keto-enol equilibria shown in Scheme 3, a cyclization steps could take place, but the product would be, in these cases, 5*H*-furan-2-one, identified by Newland et al. [8], which has also been discussed in our previous paper. Therefore, these transformations will not be discussed here (Scheme S1 in the Supplementary Material illustrates this aspect). Conversely, **D** can give origin to the product **F** directly through a ring closure step, whilst for **E** a subsequent keto-enol equilibrium is also involved (Scheme 4). In any case, the reacting system has still to overcome a non-negligible free energy barrier. In detail, from **D**, the *G* barrier to obtain **F** corresponds to 21.3 kcal mol^−1^; starting from **E**, the barrier is 21.1 kcal mol^−1^ high.

**F** has some interest because it could be seen as a reasonable candidate for the unidentified carbonyl compound: its νC=O value at B2PLYP, in conjunction with the Dunning’s basis set aug-cc-pVTZ [33] is 1842 cm^−1^. By correcting this value by the mean error defined at this computational level, 23 cm^−1^, we obtain an estimate of 1819 cm^−1^, fortuitously the same figure reported by Newland et al. [8]. However, it could appear more appropriate to restrain the pool of experimental IR frequencies to those determined in the gas phase (19 cases): this causes the mean error to be 25 cm^−1^, and moves the estimate to 1817 cm^−1^. If a similar approach is used within the other computational levels, the results obtained are as follows (in the same order): B2PLYP/cc-pVTZ: 1818 and 1818 cm^−1^. B2PLYP/aug-cc-pVDZ: 1813 and 1815 cm^−1^. Then, all of them with the cc-pVTZ basis set: M06-2X gives 1814 and 1812 cm^−1^, ωB97XD gives 1808 and 1806 cm^−1^, finally ωB97X 1809 and 1806 cm^−1^. (On the other hand, we could follow a different and maybe more daring approach, and even select just one molecule, but structurally close to **F**, γ-butyrolactone, and prefer to apply the correction to its computed B2PLYP/aug-cc-pVTZ νC=O on the basis of its available gas phase IR spectrum. The error would now drop to only 10 cm^−1^, and we would obtain 1832 cm^−1^) 

#### 2.2.2. Formation of an Anhydride

**Scheme 5:**
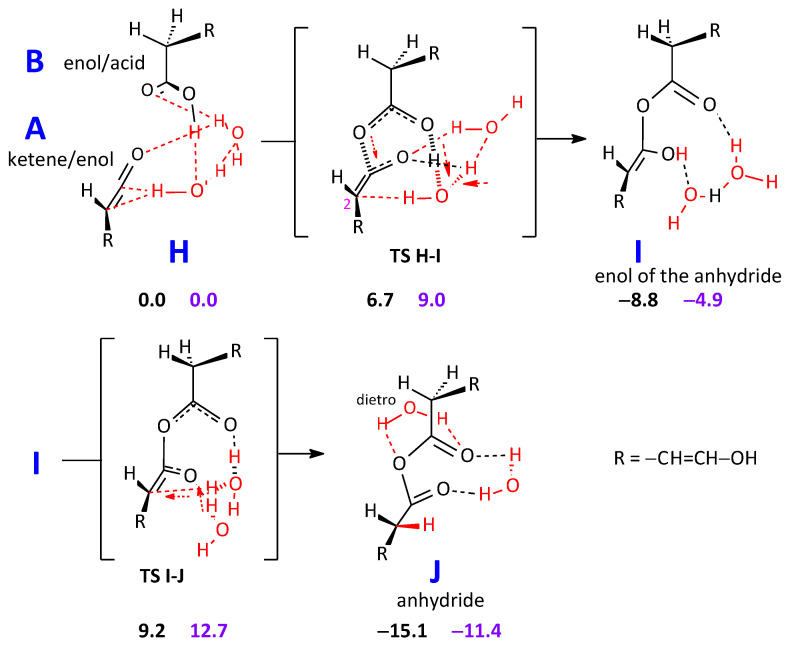
Formation of the anhydride HO–CH=CH–O.CO.O–CH=CH–OH from the nu-cleophilic attack of **B** (as carboxylic acid) to **A** (as ketene). Energy differences: black figures. Free energy differences: purple figures.

Looking for a possibly good candidate to be the unidentified carbonyl product, we have also followed a second way of forming a carbonyl compound. As said above, Kahan et al. [29], when studying the gas-phase reaction of water with ketene, observed acetic acid and acetic anhydride as products. We have thus modeled (Scheme 5, R=H) the addition of acetic acid to ketene and found a viable pathway to the anhydride via its enolic form. It is noteworthy that the nucleophilic attack by acetic acid to ketene is carried out with its carbonyl oxygen. A similar attack by its OH is not as efficient. 

When R = –CH=CH–OH, as in the real system, the enol/acid **B** conducts much in the same way a nucleophilic attack onto the central carbon of the ketene/enol moiety **A**. One overall hydrogen shift takes place from the hydroxyl of the carboxylic group in **B** to the terminal oxygen in **A**, mediated by the water molecules. Actually, three hydrogen transfers take place in the same kinetic step, because the two water molecules are fully involved in exchanging hydrogens. The intermediate product is the enol of the anhydride, **I**. The last step, leading to the anhydride **J**, presents a *G* barrier 17.6 kcal mol^−1^ high, with respect to **I**, and sees again the cooperation of the two water molecules in moving hydrogens. For **J**, two minima have been found. The vibrational data discussed below pertain to the lowest-energy one.

Our target value is, as above, 1819 cm^−1^, which corresponds to the unidentified carbonyl compound. In the case of an anhydride, the value is probably related to the higher-frequency C=O stretching mode. Unfortunately, the computation of the vibrational C=O stretching frequency by B2PLYP/aug-cc-pVTZ vibrational analysis proved unfeasible for the anhydride with R = –CH=CH–OH. Therefore, we assessed its theoretical ν_C=O_ with the smaller cc-pVTZ basis set, and the obtained highest frequency value is 1850 cm^−1^ for the C=O stretching frequencies. By taking into account the mean error at the B2PLYP/cc-pVTZ computational level, +40 cm^−1^ for 31 data (the mean error is also 40 cm^−1^ for the 25 gas phase data,) we get an estimate of 1810 cm^−1^. If we restrain the computed errors to the gas-phase data within the anhydride class, the mean error is +35 cm^−1^, and the estimate becomes 1815 cm^−1^. Then, at the B2PLYP/aug-cc-pVDZ computational level the C=O stretching frequency value 1813 cm^−1^ becomes, with a mean error of +16 cm^−1^ for 25 gas phase data, 1797 cm^−1^. If we restrain the computed errors to gas-phase data within the anhydride class a correction of 14 cm^−1^, gives an estimate of 1799 cm^−1^. Then, M06-2X gives 1817 and 1815 cm^−1^, ωB97XD 1811 and 1809 cm^−1^, ωB97X 1779 and 1779 cm^−1^, all with the cc-pVTZ basis set.

Of course, our procedure is far from perfect: reduced basis set, questionable definition of the mean error applied in the correction of the computed frequency, and a limited pool of anhydrides. Therefore, we can only offer the hypothesis that the unidentified carbonyl may be the anhydride **J**, HO–CH=CH–O.CO.O–CH=CH–OH, derived from the attack of water onto the ketene-enol intermediate. As an alternative, the intermediate **F**, i.e., 3,4*H*-5-hydroxyfuran-2-one, could seem to be a plausible hypothesis.

### 2.3. Excited States and CO Formation

We studied the mechanism of CO loss, starting from the Zee isomer of 2-butenedial because such an isomer cannot undergo the fast isomerization to ketene-enol [34]. Carbon monoxide is a common product of the photolysis of carbonyl compounds [8,11,14,35]. CO is broadly accepted to be formed in the triplet surface [14,15,35,36].

#### 2.3.1. Intersystem Crossing

Triplet states were formed after an intersystem crossing from the S_1_ state. S_2_ could be populated upon irradiation, but is expected to relax quickly to S_1_ [34,37].

The intersystem crossing rates were calculated using the Orca program. Regrettably, in Orca, meta-GGA functionals have not yet been implemented for TD-DFT gradients; therefore, we used other functionals (B3LYP [38], O3LYP [39], OLYP [40], and PBE0 [41]) to estimate the rates of the transitions between the singlet and triplet states (see Section 3). The results are summarized in Table 1 and Table 2.

**Figure 2:**
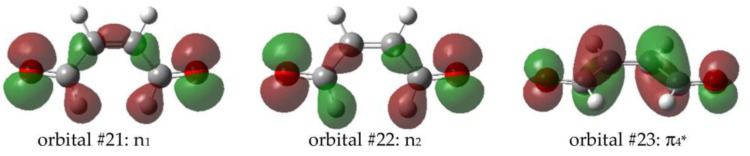
Molecular orbitals involved on the S_1_, T_1_ and T_2_ excitations.

From S_1_, the two triplet states were accessible in terms of energy. T_1_ and T_2_ both correspond to electron excitation to the π_4_* orbital from the in-phase (n_1_) and an out-of-phase (n_2_) combination of *n* orbitals (lone pairs of the oxygen atoms), Figure 2.

Both have planar geometries, but in analogy to acrolein [42,43,44], twisted geometries could also be possible. We focused on planar geometries because ISC is more favorable, and a detailed analysis of the topology of the T_1_ and T_2_ states was beyond the scope of this work.

In principle, the third triplet state (T_3_), with electronic configuration ^3^(π,π*), which lays 8.9 kcal mol^−1^ above S_1_, could be involved. The S_1_/T_3_ ISC rate was estimated at the O3LYP/cc-pVTZ level of calculation, but it was two orders of magnitude slower than the S_1_/T_1_ transition, which is 2.9 × 10^4^ s^−1^. It was ruled out, and we focused on the S_1_/T_1_ and S_1_/T_2_ intersystem crossings. These results are consistent with the findings for similar systems [37,45].

With all the tested functionals, T_1_ is about 8–11 kcal mol^−1^ more stable than S_1_ (Table 1), whereas T_2_ is only slightly more stable than S_1_ (0.3–3 kcal mol^−1^). We also tested the effect of the larger basis set aug-cc-pVTZ, but the maximum energy difference with respect to the cc-pVTZ was 0.13 kcal mol^−1^.

The intersystem crossing rates were calculated using the Orca 5.0.3 software. The intersystem crossing rate to the T_1_ state ranged from 10^6^ (OLYP, PBE0, B3LYP) to 10^8^ s^−1^ (O3LYP). The S_1_-T_2_ transition is two to four orders of magnitude faster (10^9^–10^10^ s^−1^). These values are consistent with the calculated and experimental rate of intersystem crossing for similar molecules [46,47,48].

The T_2_/T_1_ internal conversion is supposed to be very fast, because of the low barrier relevant to the MECP points, of about 1 kcal mol^−1^ or less, and consistently with results in the literature [15,48].

Lee and co-workers [49,50,51] have measured the rate of S_1_/T_1_ intersystem crossing in acetaldehyde and acetone, which ranged in the order of (3–5) × 10^8^ s^−1^. In this respect, O3LYP seems to provide a result comparable to the experiment, though the systems are different. Shinohara and Nishi predicted a similar value for acrolein [52]. However, other studies suggested that for acrolein, the rate of intersystem crossing would need to be significantly greater, 5 × 10^11^ s^−1^ [53].

More recently, Cao and Xie [45] theoretically investigated the internal conversion and intersystem crossing in some α,β-enones (acrolein and 2-cyclopentenone) using electronic structure calculations and non-adiabatic dynamics simulations. Their simulations showed that the S_1_/T_2_ transition occurs on the picosecond timescale, whereas the S_1_/T_1_ crossing is a minor channel. Very fast S_1_/T_2_ ISC was also expected during the oxidation of hydroperoxy aldehydes [47].

Inuzuca calculated the spin-orbit coupling and the conversion rate between singlet and triplets states for acrolein (3.3 × 10^8^ s^−1^), 2,4-pentadienal (4.8 × 10^9^ s^−1^), 2,4,6-heptatrienal (2.5 × 10^1^ s^−1^), and 2,4,6,8-nonatetraenal (1.4 × 10^11^ s^−1^) [46].

The photochemistry of *ortho*-nitrobenzaldehyde was studied by Voros and May [37] using MS-CASPT2 and non-adiabatic dynamics simulations. They concluded that the ISC mainly occurs from the S_1_ minimum via the S_1_/T_2_ transition. Once in the T_2_ state, rapid internal conversion T_2_/T_1_ leads to the T_1_ state. Higher singlet states (S_2_, S_3_, or S_4_) were expected to relax to S_1_ via internal conversion. The calculated ISC rate was 4.2 × 10^10^ s^−1^, similar to the experimental value 3.7 × 10^10^ s^−1^ [48].

#### 2.3.2. CO and Acrolein Formation in the T1 State

The triplet surface (T_1_) can be adequately investigated by single reference methods of calculation; therefore, we preferred to use the DFT method with the M06-2X functional according to the study of the ground state.

**Scheme 6:**
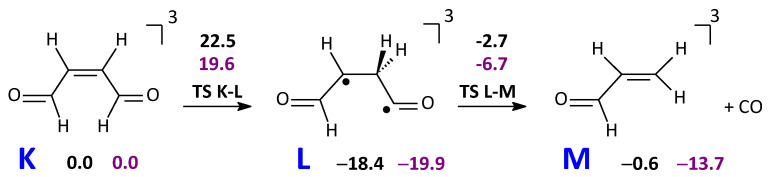
Formation of carbon monoxide from 2-butenedial on the T_1_ surface.

The reactant in its T_1_ state (**K**) is formed by intersystem crossing from S_1_/T_2_ followed by the T_2_/T_1_ crossing, analogously to other photochemical reactions [15,37]. Then, a (1,2) H transfer can occur to form a rather stable diradical intermediate **L**. The barrier for CO loss is 13.3 kcal mol^−1^ and leads to the triplet acrolein as a byproduct (Scheme 6). Acrolein has been detected in several experiments of photolysis of carbonyl compounds [7,8,11,54].

The acrolein in T_1_ state reported in the Scheme 6 has a planar geometry, but acrolein can assume several geometries, from planar to twisted, depending on the conformation of the double bond [42,43,44]. The conversion from planar ^3^(n,π*) to twisted acrolein ^3^(π,π*) is usually very fast [43]. In solution, the lifetime of the twisted acrolein is short, of the order of 10 ns, because it decays to a vibrational excited state of S_0_ via intersystem crossing [55].

We also tested C–C bond cleavage to directly form HCO and acroleyl radicals, but it was noncompetitive owing to its high energy barrier.

## 3. Materials and Methods

The DFT method, utilizing gradient procedures with the M06-2X functional [24] and the cc-pVTZ basis set [56], was employed to identify the stationary points of chemical interest on the ground state and triplet T_1_ energy hypersurface, including both minima and transition structures.

The vibrational analysis was conducted to examine the critical points’ nature. The second derivatives of the Hessian matrix were calculated analytically to determine the unscaled harmonic vibrational frequencies. Thermochemical corrections were utilized to provide estimates of the relative Gibbs free energies (*G*). The Gibbs free energy, as well as the Δ*S* term, were estimated through the total partition function, which accounts for translational, rotational, electronic, and vibrational contributions [57,58]. Δ*G* values at T = 298.15 K are reported in this paper.

The C=O stretching frequencies were calculated using the functionals M06-2X [24], ωB97X [25], ωB97XD [26], B2PLYP [27], with the cc-pVTZ basis set [56], and B2PLYP with the aug-cc-pVTZ basis set when affordable [33].

Geometry optimizations and thermochemical calculations for the ground state and the CO loss mechanism were carried out by using the GAUSSIAN16 system of programs [59]. Figure 2 has been obtained by the program GaussView [60].

The S_1_/T_n_ intersystem crossing rates were calculated by the Orca program [61,62], within the TD-DFT formalism [63,64,65]. The functionals used were: O3LYP [39], OLYP [40], PBE0 [41], and B3LYP [38], all with the cc-pVTZ basis set. The ISC rates required optimization of the S_1_ and T_n_ states, and calculation of the spin–orbit coupling and vibronic coupling (Herzberg–Teller effect) [66].

## 4. Conclusions

This paper makes reference to an interesting Euphore experimental study by Newland et al. on 2-butenedial photochemistry [8], pertinent to its chemistry in the lower tropo-sphere. It discusses in particular two different aspects of its reactions initiated by solar irradiation, a and b. 

(a) First, a series of reactions takes place on the ground state potential energy surface and involves the important ketene-enol intermediate, **A**, and water. Water was estimated to be present in a ratio ca. 100:1 with respect to ketene-enol [32]. It is also known to react with ketene under atmospheric conditions [29]. The products of water addition are intermediates bearing either a *vic*-diol or a carboxylic acid functional group (oxidation). Isomerizations and cyclizations (facilitated by water molecules) can follow, leading to a cyclic int-ermediate **F**. This molecule can be deemed a rather good candidate for the unidentified carbonyl product observed by Newland et al., because its carbonyl vibrational frequency is estimated to be quite close to the value measured in the experimental study, depending on the correction applied to the computed frequency. However, this seems to be rather energy demanding facing free energy barriers somewhat higher than 20 kcal mol^−1^. An alternative pathway, also initiated by water intervention, then assisted by it, produces the anhydride **J**, derived from the attack of water onto the ketene-enol intermediate **A**. Though its estimated carbonyl stretching frequency is almost as close as that of **F** to the target value, the highest *G* barrier encountered is somewhat lower than 18 kcal mol^−1^. We can therefore put forth the twofold hypothesis that the unidentified carbonyl may be either the anhydride **J**, HO–CH=CH–O.CO.O–CH=CH–OH, or, alternatively, the product **F**, i.e., 3,4*H*-5-hydroxyfuran-2-one.

(b) The second aspect dealing with butenedial photochemistry is the possible involvement of its most accessible singlet and triplet excited states. S_1_ has a central role. Having ruled out any role of T_3_, based on calculated intersystem crossing rates, both T_1_ and T_2_ result in accessibility in terms of energy, but the S_1_/T_2_ transition is significantly faster than S_1_-T_1_. However, T_2_/T_1_ internal conversion populates the lowest triplet state. On this surface, butenedial undergoes a hydrogen shift by overcoming a *G* barrier of less than 20 kcal mol^−1^. The photolytic two-step reaction produces carbon monoxide and triplet propenal.

## Figures and Tables

**Table 1:** T_1_ and T_2_ energy minima relative to the S_1_ minimum in kcal mol^−1^.

Basis Set	cc-pVTZ		aug-cc-pVTZ	
Functional	T_1_	T_2_	T_1_	T_2_
O3LYP	−8.78	−0.37	−8.67	−0.32
OLYP	−8.18	−1.95	−8.05	−1.90
PBE0	−10.72	−2.85	−10.60	−2.72
B3LYP	−9.90	−2.99	−9.77	−2.86

**Table 2:** ISC rates in s^−1^, and MECP barriers from T_2_, in kcal mol^−1^.

Functional ^1^	S_1_/T_1_ ISC Rate	S_1_/T_2_ ISC Rate	T_2_/T_1_ MECP Barrier
O3LYP	8.7 × 10^7^	1.0 × 10^9^	0.85
OLYP	1.6 × 10^6^	1.1 × 10^10^	1.01
PBE0	5.9 × 10^6^	1.2 × 10^10^	0.99
B3LYP	6.9 × 10^6^	1.6 × 10^10^	0.81

^1^ cc-pVTZ basis set.

## Data Availability

The data presented in this study are available in article and Supplementary Materials.

## Supplementary Materials

The following supporting information can be downloaded at: https://www.mdpi.com/article/10.3390/molecules29030575/s1. Supplementary Materials for this article include the geometries and energetics of the optimized structures, and scheme with ruled out mechanisms.
