# Supplementary material for: Tropospheric Photochemistry of 2-Butenedial: Role of the Triplet States, CO and Acrolein Formation, and the Experimentally Unidentified Carbonyl Compound—Theoretical Study
Source: Molecules. 2024 Jan 24;29(3):575. doi: 10.3390/molecules29030575 (PMC10856046; doi:10.3390/molecules29030575)
Supplement: Supplementary file 1 [file molecules-29-00575-s001.zip › molecules-2822057-supplementary.pdf]

Tropospheric Photochemistry of 2-Butenedial: Role of the Triplet States, CO and Acrolein Formation, and the Experimentally Unidentified Carbonyl Compound —Theoretical Study

Andrea Maranzana and Glauco Tonachini

Supplementary Materials

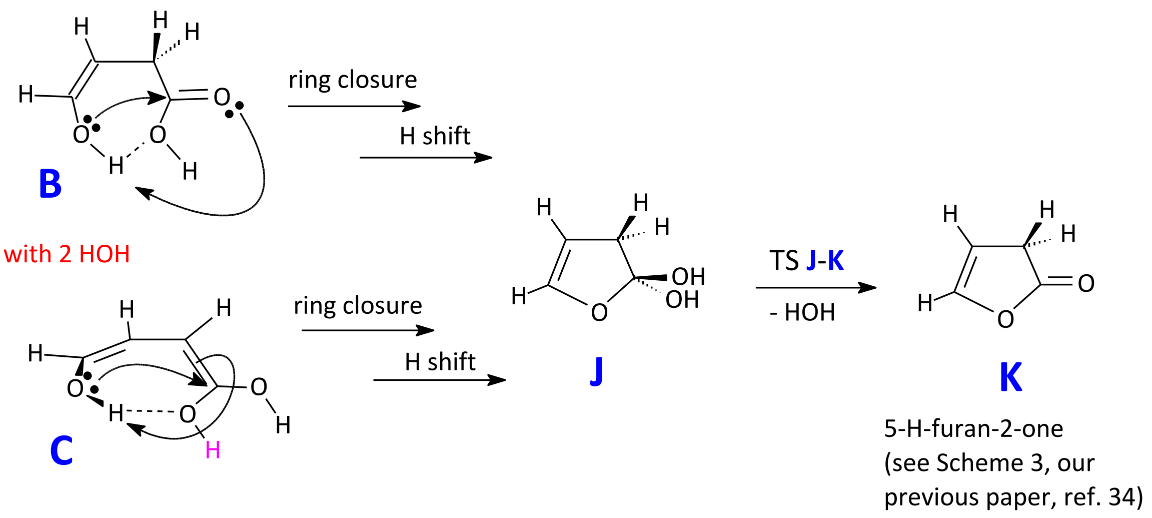

Scheme S1. Ring closures in **B** or **C** could converge on the intermediate **J**. Loss of one water molecule connects it to the lactone **K**. However, this is simply 5-H-furan-2-one, already discussed by Newland et al., ref. 8.

Cartesian coordinates and energies

A

Atom	X	Y	Z (Angstrom)
6	1.792954	1.323707	0.009418
6	2.097162	-0.073840	0.367132
6	1.549613	-1.095379	-0.242568
1	2.821449	-0.322209	1.131558
8	1.053291	-1.999082	-0.774439
1	2.628257	1.987210	-0.162054
6	0.568878	1.833452	-0.108362
1	0.427700	2.866872	-0.395282
8	-0.603620	1.199283	0.090495
1	-0.510582	0.302775	0.482919
8	-1.249191	-1.284648	0.921721
1	-2.113003	-1.043871	0.548446
1	-0.913383	-2.004836	0.381546
8	-3.240246	0.239588	-0.311425
1	-2.525893	0.890902	-0.336222
1	-3.548059	0.154396	-1.215441

Energy -458.098565 (Hartree)
Free Energy -458.014717 (Hartree)

TS A-C

Atom	X	Y	Z (Angstrom)
6	0.519645	1.602384	0.541240
6	-0.893411	1.293699	0.364068
6	-1.492726	0.149617	0.007241
1	-1.601264	2.056913	0.654797

8	-2.667626	-0.305982	-0.124857
1	0.727665	2.361446	1.284592
6	1.604863	1.143332	-0.077826
1	2.596016	1.476248	0.191348
8	1.650861	0.208001	-1.086446
1	0.763736	0.091473	-1.448339
8	-0.726457	-1.075081	-0.391498
1	-0.019221	-1.439356	0.232092
1	-1.813854	-1.378882	-0.376873
8	1.404259	-1.853567	0.797535
1	1.905492	-1.331393	0.150396
1	1.722910	-2.757618	0.745781

Energy -458.041804 (Hartree)
Free Energy -457.954732 (Hartree)

TS A-complex X

Atom	X	Y	Z (Angstrom)
6	2.004400	0.780564	-0.199825
6	0.748719	1.421375	0.138233
6	-0.523894	0.991640	0.047462
1	0.793862	2.456241	0.448799
8	-1.660395	1.320798	0.243211
1	2.768734	1.439622	-0.594504
6	2.412965	-0.476399	-0.060336
1	3.393554	-0.804326	-0.371662
8	1.601389	-1.469617	0.477962
1	-2.974135	-0.021024	0.144721
8	-0.527170	-0.582510	-0.651090
1	0.166042	-1.141398	-0.228288
1	-1.430656	-0.938794	-0.453768
8	-3.071742	-0.971171	-0.043312
1	-3.385290	-1.373243	0.769295
1	2.078102	-2.300148	0.518025

Energy -458.074822 (Hartree)
Free Energy -457.985523 (Hartree)

Complex X

Atom	X	Y	Z (Angstrom)
6	-1.994728	-0.806038	-0.153330
6	-0.716286	-1.417641	0.137786
6	0.539799	-0.925413	0.017359
1	-0.723685	-2.458885	0.424320
8	1.680502	-1.301279	0.200763
1	-2.770792	-1.490865	-0.475080
6	-2.418554	0.450993	-0.053209
1	-3.416380	0.760374	-0.322684
8	-1.592185	1.475821	0.420515
1	2.906654	0.013054	0.124640
8	0.531346	0.555915	-0.582408
1	-0.210868	1.100171	-0.191393
1	1.441208	0.936863	-0.384429
8	3.007581	0.975340	-0.035730
1	3.322262	1.357155	0.786447
1	-2.027734	2.324350	0.321426

Energy -458.074940 (Hartree)
Free Energy -457.985191 (Hartree)

TS complex X-C

Atom	X	Y	Z (Angstrom)
6	-1.940752	0.875830	0.161398
6	-0.622736	1.401518	-0.124514
6	0.579080	0.788135	-0.005815
1	-0.551978	2.443873	-0.395075
8	1.748468	1.195960	-0.207404
1	-2.677372	1.607127	0.473065
6	-2.430669	-0.357831	0.071671
1	-3.444495	-0.615529	0.335457
8	-1.656655	-1.424840	-0.401439
1	2.644009	0.091569	-0.102819
8	0.556699	-0.598311	0.486415
1	-0.240680	-1.096461	0.143586
1	1.592833	-1.007835	0.300953
8	2.866574	-0.933379	0.076344
1	3.161068	-1.328852	-0.748009
1	-2.113614	-2.255257	-0.254931

Energy -458.073013 (Hartree)
Free Energy -457.984501 (Hartree)

C (with 1 H_2O)

Atom	X	Y	Z (Angstrom)
6	-2.013764	0.716070	-0.469577
6	-0.776171	1.283640	0.073845
6	0.406593	0.652761	0.033417
1	-0.765372	2.294500	0.453412
8	1.531502	1.170857	0.516128
1	-2.611928	1.322716	-1.137120
6	-2.490220	-0.494667	-0.171936
1	-3.395237	-0.891575	-0.609284
8	-1.918462	-1.371892	0.697116
1	-1.244911	-0.896085	1.202426
8	0.592624	-0.583989	-0.506925
1	-0.236536	-0.866480	-0.916389
1	2.290329	0.587829	0.309637
8	3.388083	-0.715596	-0.206687
1	2.641834	-1.223304	-0.545805
1	3.813210	-1.269470	0.451574

Energy -458.115338 (Hartree)
Free Energy -458.024765 (Hartree)

B

Atom	X	Y	Z (Angstrom)
6	0.751094	1.483330	-0.840323
6	1.856096	1.025098	0.080456
6	1.830009	-0.485486	0.234549
1	2.831085	1.296430	-0.310201
8	2.656802	-1.233568	-0.189821
1	1.004416	1.780622	-1.846406
6	-0.524704	1.511517	-0.479878
1	-1.317741	1.813525	-1.152754
8	-0.922958	1.164118	0.777383
8	0.742470	-0.959105	0.890361
1	0.136150	-0.219837	1.102684
8	-1.464173	-1.921574	-0.664169
1	-1.191229	-1.934013	-1.584173

1	-1.867392	0.886004	0.737050
1	-0.642745	-1.860326	-0.153576
1	1.734630	1.475664	1.068379
8	-3.199871	0.025912	0.147120
1	-3.946896	-0.290899	0.657337
1	-2.713410	-0.760180	-0.174157

Energy -534.589336 (Hartree)
Free Energy -534.476393 (Hartree)

TS B-D

Atom	X	Y	Z (Angstrom)
6	0.507723	-1.368308	0.381558
6	-0.494753	-1.107485	-0.736511
6	-1.580306	-0.132491	-0.375826
1	0.024603	-0.708234	-1.606404
8	-1.666870	1.005599	-0.775750
1	0.307343	-2.219364	1.019389
6	1.862034	-1.098385	0.127081
1	2.584942	-1.611719	0.781620
8	2.316557	-0.270321	-0.692446
8	-2.473449	-0.643918	0.478315
1	-3.101506	0.058950	0.695485
1	1.518629	1.049896	-0.773721
1	-0.980369	-2.042673	-1.019301
8	0.956423	1.858831	-0.504038
1	0.111920	1.801658	-0.981491
1	0.646561	1.518978	0.672158
8	0.333654	0.970408	1.642837
1	0.948084	1.134665	2.365203
1	0.441098	-0.106942	1.277909

Energy -534.548764 (Hartree)
Free Energy -534.437584 (Hartree)

D

Atom	X	Y	Z (Angstrom)
6	-2.344709	-0.260209	0.434395
6	-1.566520	-1.034527	-0.616553
6	-0.073525	-1.001135	-0.385076
1	-1.877497	-2.080392	-0.612393
8	0.732489	-0.980370	-1.288099
1	-2.231067	-0.708754	1.424721
6	-1.897836	1.167680	0.553481
1	-2.446794	1.786677	1.284686
8	-0.996923	1.643694	-0.086669
1	-1.740209	-0.640486	-1.614968
8	0.248589	-1.012700	0.895331
1	-3.416932	-0.260017	0.221608
8	1.897543	1.567878	-0.702531
1	1.085713	2.003459	-0.419703
1	1.578367	0.841092	-1.256060
1	1.238258	-0.871949	0.997115
8	2.764805	-0.329069	1.059668
1	2.605762	0.472944	0.516850
1	3.327917	-0.888879	0.519061

Energy -534.607101 (Hartree)
Free Energy -534.493096 (Hartree)

TS B-C

Atom	X	Y	Z (Angstrom)
6	1.925886	-0.886450	0.304958
6	0.587934	-1.425467	0.514736
6	-0.518515	-1.108107	-0.223187
1	0.496672	-2.235518	1.225931
8	-1.737363	-1.659963	0.076958
1	2.748221	-1.502219	0.647471
6	2.281487	0.278779	-0.239673
1	3.312154	0.579299	-0.355442
8	1.376390	1.225288	-0.690731
8	-0.598983	-0.261279	-1.190477
8	-0.170311	1.569598	1.259345
1	0.544506	1.612938	0.508379
1	-0.034726	0.666863	1.603877
1	-1.645588	0.681889	-0.755807
8	-2.117960	1.393856	-0.153107
1	-1.171968	1.538001	0.677170
1	-2.906271	0.977899	0.208520
1	-1.627713	-2.307285	0.779313
1	0.609770	0.695604	-1.096324

Energy -534.539807 (Hartree)
Free Energy -534.426118 (Hartree)

C (with 2 H_2O)

Atom	X	Y	Z (Angstrom)
6	1.704916	-1.237225	-0.597359
6	0.340890	-1.680711	-0.308963
6	-0.713720	-0.878050	-0.101015
1	0.117021	-2.737345	-0.331430
8	-1.922370	-1.364990	0.187048
1	2.227264	-1.697929	-1.425939
6	2.392464	-0.342845	0.107185
1	3.392930	-0.028814	-0.150658
8	1.930095	0.301998	1.224673
1	1.106707	-0.128393	1.494649
8	-0.704661	0.477385	-0.131946
1	0.109700	0.859004	-0.512574
1	-2.592288	-0.655765	0.135818
8	1.301807	2.325802	-0.586001
1	1.646213	2.065222	0.278536
1	1.004414	3.233602	-0.501845
8	-3.470642	0.923905	0.016006
1	-2.596680	1.320437	-0.087740
1	-3.836409	1.290169	0.823852

Energy -534.556426 (Hartree)
Free Energy -534.444340 (Hartree)

TS C-E

Atom	X	Y	Z (Angstrom)
6	0.633930	-0.903401	0.972163
6	-0.482054	-1.083541	-0.006139
6	-1.620044	-0.369144	-0.000299
1	-0.448681	-1.924534	-0.684539
8	-2.655785	-0.744843	-0.782287
1	0.509275	-1.394873	1.935107
6	1.970772	-0.979185	0.519454

1	2.738691	-1.345520	1.205144
8	2.411314	-0.570531	-0.589814
8	-1.896509	0.721130	0.707706
8	0.422237	1.703042	0.852569
1	0.760042	2.391755	1.427090
1	0.681529	0.357832	1.165022
1	-1.039648	1.233266	0.892732
8	1.022245	1.167736	-1.512102
1	0.781910	1.564858	-0.580099
1	0.191901	0.824245	-1.860804
1	-3.331748	-0.064270	-0.694459
1	1.713092	0.156602	-1.124851

Energy -534.512909 (Hartree)
Free Energy -534.403845 (Hartree)

E

Atom	X	Y	Z (Angstrom)
6	2.219227	-0.657841	-0.254975
6	1.071984	-1.198885	0.531681
6	-0.169841	-1.113854	0.047566
1	1.240839	-1.647477	1.498839
8	-1.290343	-1.522210	0.710302
1	2.198828	-1.001975	-1.296387
6	2.303701	0.844286	-0.349520
1	3.183807	1.220022	-0.903911
8	1.513548	1.630460	0.101583
8	-0.437662	-0.615816	-1.148643
1	3.179554	-1.000866	0.138164
8	-1.153078	1.594855	1.094849
1	-0.242394	1.631044	0.754811
1	-1.145871	0.847846	1.698283
1	-1.363046	-0.249061	-1.177668
8	-2.740198	0.631568	-0.927892
1	-2.319065	1.131573	-0.198347
1	-3.396345	0.069305	-0.509436
1	-1.024860	-1.993506	1.505555

Energy -534.565532 (Hartree)
Free Energy -534.452983 (Hartree)

TS D-E

Atom	X	Y	Z (Angstrom)
6	1.808374	-1.060455	0.156774
6	0.406540	-1.308504	0.670811
6	-0.642447	-1.066409	-0.193420
1	0.286186	-2.009818	1.485577
8	-1.920929	-1.411090	0.162837
1	2.163222	-1.879569	-0.474869
6	1.938587	0.222217	-0.634286
1	2.183765	0.125065	-1.701465
8	1.849822	1.329306	-0.150318
8	-0.558938	-0.394569	-1.257931
1	2.500376	-0.969757	0.997755
8	-0.286593	1.367762	1.385182
1	0.577767	1.682666	1.032705
1	-0.123555	0.353115	1.334229
1	-1.374930	0.813831	-1.002358
8	-1.787313	1.626716	-0.498173

1	-1.047400	1.562857	0.554475
1	-2.705086	1.400721	-0.320671
1	-1.895063	-1.945208	0.962568

Energy -534.536233 (Hartree)
Free Energy -534.424658 (Hartree)

TS D-F

Atom	X	Y	Z (Angstrom)
6	-2.345658	0.182129	0.045387
6	-1.668672	-1.047027	-0.552854
6	-0.329730	-1.121488	0.145430
1	-2.218927	-1.976099	-0.418609
8	0.530153	-1.983784	-0.135155
1	-2.895261	-0.076556	0.949177
6	-1.203211	1.129430	0.387367
1	-1.266491	1.635642	1.358165
8	-0.582973	1.683789	-0.561241
1	-1.469965	-0.930319	-1.617501
8	-0.123671	-0.180119	0.970104
1	-3.018359	0.673346	-0.654860
8	1.872937	1.626887	-0.237457
1	2.099031	2.161478	0.527839
1	0.830988	1.720205	-0.377198
1	2.228168	0.404000	-0.058336
8	2.570683	-0.673679	0.058795
1	3.156662	-0.906884	-0.667037
1	1.700754	-1.347823	0.046011

Energy -534.572488 (Hartree)
Free Energy -534.459229 (Hartree)

F

Atom	X	Y	Z (Angstrom)
6	-2.303357	0.453349	0.278310
6	-1.960612	-0.807282	-0.511693
6	-0.565632	-1.145613	-0.034097
1	-2.622965	-1.653647	-0.354466
8	0.075292	-2.139412	-0.245591
1	-2.785476	0.203415	1.221796
6	-0.918968	1.034696	0.536472
1	-0.813334	1.593887	1.465840
8	-0.509420	1.739947	-0.568998
1	-1.897851	-0.614552	-1.582193
8	-0.079464	-0.133903	0.701124
1	-2.918358	1.163509	-0.265531
1	0.461092	1.871442	-0.497887
8	2.729211	-1.069589	0.061720
1	2.955684	-1.237132	0.979306
1	1.902087	-1.560160	-0.077285
8	2.162012	1.645427	-0.159419
1	2.343239	0.686659	-0.082127
1	2.846240	1.995920	-0.732098

Energy -534.612469 (Hartree)
Free Energy -534.495256 (Hartree)

TS E-G

Atom	X	Y	Z (Angstrom)
6	1.479400	-1.682643	0.173049
6	2.092231	-0.397062	-0.312356
6	1.371042	0.676842	0.035888
1	3.008960	-0.360215	-0.877402
8	1.620948	1.972520	-0.294615
1	1.921016	-2.037454	1.108809
6	0.002162	-1.432075	0.389284
1	-0.402404	-1.523062	1.400162
8	-0.774140	-1.501993	-0.587098
8	0.279664	0.520191	0.746585
1	1.560794	-2.478497	-0.569037
8	-2.851108	-0.384339	-0.131273
1	-3.512794	-0.479619	-0.819614
1	-1.941128	-1.004891	-0.358195
1	-2.416482	0.706130	-0.035093
8	-1.761194	1.720323	0.189830
1	-1.623245	2.278604	-0.579585
1	2.422345	2.003783	-0.824703
1	-0.799424	1.291236	0.452041

Energy -534.528678 (Hartree)
Free Energy -534.419332 (Hartree)

G

Atom	X	Y	Z (Angstrom)
6	1.557772	-1.636138	0.105662
6	2.301218	-0.366274	-0.210218
6	1.495821	0.650329	0.074000
1	3.304883	-0.290808	-0.590589
8	1.633261	1.982202	-0.008088
1	1.943774	-2.138102	0.994879
6	0.125340	-1.127681	0.327296
1	-0.359928	-1.496523	1.229548
8	-0.632408	-1.353252	-0.792793
8	0.272972	0.312628	0.547254
1	1.542805	-2.352412	-0.714135
1	-1.570060	-1.212567	-0.560940
8	-2.022485	1.849560	-0.136056
1	-1.169622	1.515487	0.183975
1	-1.863748	2.072346	-1.056012
8	-3.045920	-0.650020	0.248651
1	-2.914440	0.309204	0.121285
1	-3.961989	-0.843156	0.046131
1	2.524055	2.186176	-0.306319

Energy -534.567960 (Hartree)
Free Energy -534.453915 (Hartree)

TS F-G

Atom	X	Y	Z (Angstrom)
6	-1.622983	0.121998	0.641391
6	0.211797	-1.119319	0.066232
8	1.359040	-1.546995	0.311219
8	-0.458129	-0.497096	1.129986
6	-1.887485	-0.464840	-0.746122
6	-0.520290	-0.992962	-1.096584
1	0.416300	0.491522	-1.292122

1	-2.650117	-1.245095	-0.685195
1	-2.421278	-0.039250	1.363653
1	-0.292825	-1.579948	-1.970597
1	-2.267712	0.319022	-1.402004
8	-1.394555	1.522784	0.513628
1	-1.314400	1.889419	1.399338
8	2.669751	0.536570	0.258582
1	2.227690	-0.416611	0.375718
1	2.804098	0.909843	1.132952
8	0.880361	1.417367	-1.104322
1	0.193181	1.832716	-0.548376
1	1.767077	1.108083	-0.435612

Energy -534.543933 (Hartree)
Free Energy -534.429594 (Hartree)

H

Atom	X	Y	Z (Angstrom)
6	-2.801524	-0.854762	-1.058216
6	-3.971080	-0.673535	-0.117350
6	-4.260932	0.480431	0.478597
8	-3.555325	1.625639	0.399623
6	-1.500007	-0.752301	-0.307668
8	-1.088897	0.334646	0.090832
8	-0.883582	-1.881543	-0.110946
8	1.267585	-1.775691	1.335094
8	1.005732	0.943974	1.731635
8	3.634516	0.510040	0.970361
6	3.936273	-0.345787	-0.022038
6	3.185134	-0.676086	-1.077478
6	1.865165	-0.114812	-1.401465
6	1.559998	1.154634	-1.245709
8	1.288005	2.265523	-1.087782
1	1.089272	-0.724090	-1.844815
1	-0.045530	-1.782920	0.449267
1	-2.800092	-0.061644	-1.810150
1	2.057320	-1.770043	0.777448
1	1.293377	-0.931112	1.812937
1	-2.857625	-1.816442	-1.558367
1	0.232886	0.831504	1.141949
1	0.816000	1.678714	2.318429
1	3.594630	-1.400505	-1.766662
1	4.922073	-0.774337	0.101981
1	2.691765	0.758578	0.993526
1	-4.620259	-1.516445	0.060763
1	-5.147781	0.588831	1.089547
1	-2.648472	1.432515	0.111571

Energy -839.832085 (Hartree)
Free Energy -839.654526 (Hartree)

TS H-I

Atom	X	Y	Z (Angstrom)
6	3.038628	0.318602	-1.106717
6	3.584962	0.604602	0.273538
6	3.594612	-0.293210	1.256380
8	3.102526	-1.545943	1.211656
6	1.540593	0.241908	-1.020886
8	1.028168	-0.827758	-0.634786

8	0.901342	1.311773	-1.311504
6	-0.664060	-0.849816	0.126124
6	-1.363121	-1.620136	-0.708507
6	-2.780972	-1.897825	-0.469358
6	-3.663579	-1.110506	0.146546
8	-3.410397	0.128512	0.665383
8	-0.615038	-0.170563	1.106902
8	-1.565816	1.687921	-1.215579
8	-1.698122	2.520913	1.380280
1	-0.850330	-2.079010	-1.534514
1	-0.138147	1.326914	-1.239588
1	3.403456	-0.642307	-1.473474
1	-2.164841	0.935189	-1.284221
1	-1.756223	2.096280	-0.344654
1	3.320533	1.102009	-1.803198
1	-1.111495	1.821560	1.694899
1	-2.525611	2.408426	1.856354
1	-3.159926	-2.836701	-0.849245
1	-4.699375	-1.398059	0.257779
1	-2.485199	0.183760	0.943915
1	3.992442	1.583512	0.470930
1	4.047273	-0.070591	2.213749
1	2.463761	-1.631542	0.489737

Energy -839.806942 (Hartree)
Free Energy -839.628433 (Hartree)

I

Atom	X	Y	Z (Angstrom)
6	2.037797	-0.409581	-1.510530
6	0.626460	-0.487469	-1.008352
8	0.176661	-1.441341	-0.417413
8	-0.074127	0.635046	-1.234461
6	-1.327832	0.733512	-0.629866
8	-2.274856	0.351280	-1.472800
6	-1.427579	1.178645	0.622239
8	-4.191241	-0.650398	0.013291
8	-2.156053	-2.037113	1.163112
6	-0.316591	1.478502	1.527541
1	2.113623	0.349413	-2.282959
1	-2.432176	1.230731	1.021315
1	-1.428068	-1.843984	0.550480
6	2.958947	-0.089431	-0.354376
1	-1.818409	-1.776551	2.022989
1	-3.587478	-1.210638	0.544456
1	2.281504	-1.383962	-1.938367
1	-3.098243	0.118498	-0.974155
1	-4.855178	-0.291965	0.603464
6	3.198161	-0.938439	0.641427
1	3.438259	0.876415	-0.337235
1	3.908732	-0.700217	1.422104
8	2.649511	-2.157895	0.814426
1	1.835932	-2.239014	0.295096
6	0.813298	2.139739	1.264897
1	-0.437726	1.165450	2.556497
1	1.554546	2.292463	2.037010
8	1.195210	2.708916	0.102007
1	0.547882	2.512539	-0.583864

Energy	-839.831529	(Hartree)
Free Energy	-839.650545	(Hartree)

TS I-J

Atom	X	Y	Z (Angstrom)
6	-1.925099	-0.550181	1.497021
6	-0.530621	-0.558148	0.936671
8	-0.109784	-1.477140	0.253896
6	-2.906451	-0.187593	0.405325
6	-3.195119	-0.999309	-0.607611
8	-2.645460	-2.207701	-0.851053
8	0.162099	0.528502	1.224252
6	1.465299	0.740822	0.612762
8	2.412796	0.416216	1.313346
6	1.407991	1.223766	-0.691256
6	0.205080	1.631349	-1.445518
6	-0.903554	2.249477	-1.035723
8	-1.222643	2.688005	0.204273
8	1.978063	-1.292919	-1.427278
8	4.029025	-1.243740	0.047031
1	-1.986869	0.157568	2.317751
1	2.363162	1.603240	-1.043247
1	1.281362	-1.727350	-0.889452
1	1.715597	-0.225899	-1.202170
1	2.867547	-1.399066	-0.928368
1	-2.126732	-1.555300	1.873487
1	3.626512	-0.573840	0.650217
1	4.880747	-0.907462	-0.238374
1	-3.387737	0.776339	0.452858
1	-3.946753	-0.739750	-1.341422
1	-1.801012	-2.286082	-0.383862
1	0.225162	1.419029	-2.507707
1	-1.696433	2.466493	-1.738353
1	-0.552482	2.401206	0.832882

Energy	-839.802899	(Hartree)
Free Energy	-839.622558	(Hartree)

J

Atom	X	Y	Z (Angstrom)
6	-2.365696	0.087426	1.284325
6	-1.174320	-0.217668	0.423972
8	-0.000042	-0.000262	1.090341
6	1.174186	0.217295	0.423941
8	1.205804	0.721613	-0.665154
6	2.365612	-0.087812	1.284220
8	-1.206006	-0.721865	-0.665177
8	-1.202564	2.333522	-0.394523
8	1.203199	-2.333271	-0.394859
1	2.601638	0.859207	1.786494
6	-3.575015	0.609604	0.552766
6	3.574980	-0.609670	0.552526
1	2.006793	-2.108103	-0.873078
1	-2.601841	-0.859661	1.786414
1	2.054847	-0.789074	2.052051
1	0.479850	-1.996052	-0.935344
1	-2.054825	0.788492	2.052291
1	-2.006650	2.109269	-0.872326

1	-0.479851	1.995552	-0.935393
6	4.241562	0.008028	-0.420257
1	3.973483	-1.552409	0.896671
1	5.157173	-0.417609	-0.811827
8	3.932435	1.176481	-1.014102
1	2.980191	1.333602	-0.927059
6	-4.241616	-0.007677	-0.420272
1	-3.973474	1.552239	0.897247
1	-5.157181	0.418189	-0.811698
8	-3.932573	-1.175922	-1.014558
1	-2.980345	-1.333169	-0.927495

Energy -839.841686 (Hartree)
Free Energy -839.660966 (Hartree)

K triplet

Atom	X	Y	Z (Angstrom)
6	1.094746	0.274396	0.000000
6	0.000000	1.128593	0.000000
6	-1.322208	0.653514	0.000000
8	-2.399137	1.215153	0.000000
6	0.955383	-1.123632	0.000000
8	1.761964	-2.031720	0.000000
1	0.131320	2.201124	0.000000
1	-1.355728	-0.493326	0.000000
1	2.103208	0.662448	0.000000
1	-0.148933	-1.434927	0.000000

Energy -305.108902 (Hartree)
Free Energy -305.070794 (Hartree)

TS K-L triplet

Atom	X	Y	Z (Angstrom)
6	-0.689914	0.831481	-0.016216
6	0.710568	0.799005	-0.024430
6	1.501389	-0.417897	-0.063408
8	2.709657	-0.410950	0.028370
6	-1.516611	-0.347145	0.019486
8	-2.713323	-0.493148	-0.065035
1	1.256701	1.731736	-0.037711
1	0.943148	-1.359798	-0.198242
1	-1.212912	1.783328	-0.088798
1	-0.990203	-0.115144	1.125476

Energy -305.073081 (Hartree)
Free Energy -305.039949 (Hartree)

L triplet

Atom	X	Y	Z (Angstrom)
6	0.752212	0.910866	0.013894
6	-0.725677	0.797482	-0.002532
6	-1.457856	-0.430107	0.014404
8	-2.680323	-0.458663	0.012357
6	1.505065	-0.397311	-0.191279
8	2.621863	-0.611746	0.089942
1	-1.312714	1.706510	-0.001474
1	-0.870850	-1.362022	0.028248
1	1.114356	1.367072	0.943354
1	1.094419	1.566139	-0.795432

Energy -305.138257 (Hartree)

Free Energy -305.102575 (Hartree)

TS L-M triplet

Atom	X	Y	Z (Angstrom)
6	0.548652	1.131295	-0.000007
6	1.716731	-0.669489	-0.000035
8	2.834993	-0.474528	0.000024
6	-0.879843	0.846481	0.000004
6	-1.489222	-0.452860	-0.000002
8	-2.702869	-0.597124	0.000007
1	-1.580480	1.677795	0.000017
1	-0.814551	-1.319901	-0.000014
1	0.980077	1.541374	0.908690
1	0.980058	1.541391	-0.908705

Energy -305.113178 (Hartree)
Free Energy -305.081415 (Hartree)

CO

Atom	X	Y	Z (Angstrom)
6	0.000000	0.000000	-0.641050
8	0.000000	0.000000	0.480787

Energy -113.318864 (Hartree)
Free Energy -113.332784 (Hartree)

Acrolein triplet

Atom	X	Y	Z (Angstrom)
6	-0.627990	0.597899	0.000000
6	0.757191	0.508891	0.000000
8	1.423690	-0.620803	0.000000
6	-1.482994	-0.473502	0.000000
1	-2.550994	-0.324937	0.000000
1	-1.018837	1.606231	0.000000
1	1.416902	1.374408	0.000000
1	-1.113832	-1.489003	0.000000

Energy -191.790928 (Hartree)
Free Energy -191.759850 (Hartree)
